# Optimization of synergic antibacterial activity of *Punica granatum* L. and *Areca nut* (P.G.L.A.N) extracts through response surface methodology

**DOI:** 10.1038/s41598-023-32900-1

**Published:** 2023-04-13

**Authors:** Parvin Gharbani, Neda Jam, Hossein Doshmanfekan, Ali Mehrizad

**Affiliations:** 1 Department of Chemistry, Ahar Branch, Islamic Azad University, Ahar, Iran; 2 Industrial Nanotechnology Research Center, Tabriz Branch, Islamic Azad University, Tabriz, Iran; 3 Department of Chemical Engineering, Ahar Branch, Islamic Azad University, Ahar, Iran; 4 Department of Chemistry, Tabriz Branch, Islamic Azad University, Tabriz, Iran

**Keywords:** Biochemistry, Microbiology, Health care, Pathogenesis

## Abstract

The primary objective of this study was to evaluate the use of natural compounds as opposed to chemical preservatives. This study employed response methodology to evaluate the synergistic antibacterial effect of *Areca nut* and *Punica granatum* L. extract. Independent variables included extract type (*Punica granatum* L., *Areca nut*, and their mixture), solvent (water, ethanol, methanol), bacterial type (*S. aureus*, *Salmonella*, *E. coli*), and extract concentration (1, 10, 100 mg/L). The sensitivity was determined using the disk diffusion method, and the diameter of the inhibitory zone was measured. On the specified bacteria, the MIC (minimum inhibitory concentration) and MBC (minimum bactericidal concentration) of each extract were ascertained using the serial dilution method. This study revealed the existence of beneficial synergistic effects between the two extracts. Results indicated that the ethanolic extracts of *Punica granatum* L. and *Areca nut* had a synergistic effect on *E. coli*.

## Introduction

Food safety and health are interdependent. Unsafe food perpetuates a cycle of disease and malnutrition. Therefore, improving food safety is essential for achieving sustainable development goals^1^. According to the World Health Organization (WHO), approximately one in ten people in the world become ill after consuming contaminated food, resulting in an annual death toll of 420,000. In the food industry, modern preservation techniques, such as using antioxidants, are of particular interest^2^. The addition of antioxidants is one of the most effective methods for preventing food spoilage. This technique is commonly used to extend the shelf life of food, improve the stability of fats and fatty foods, and thereby prevent the loss of sensory properties and nutritional value^3^. Numerous reports have been published thus far on the antimicrobial activity of plants. Polyphenols, alkaloids, terpenes, and coumarins play a significant role in the effect of plant extract on microbial growth^4^. The search for bio-metabolites to inhibit biodegradation and lengthen the shelf life of perishable products has attracted growing interest. In this regard, the emphasis is primarily on plant extracts and metabolites derived from microorganisms, or their combined use, to enhance antimicrobial activity^5^. Currently, due to the mutagenicity, toxicity, and carcinogenicity of synthetic antioxidants, the use of natural antioxidants such as polyphenols and vitamins with antioxidant properties such as ascorbic acid, beta carotene, vitamin A, and plant extracts has increased. They exhibit protective effects against chronic diseases, including cancer, diabetes, cardiovascular disease, Alzheimer’s, cataracts, and mutagenicity^6^. Therefore, controlling these bacteria in food is essential. In addition, by causing these bacteria to develop resistance to preservatives, their effectiveness against bacteria is diminished, which contributes to the spread of disease and endangers the health of society. Therefore, it is essential to identify resistant strains and propose methods for their control in food^7,8^.

Rahman et al. compared the antibacterial activity of *Areca catechu* nut extracted with various solvents and observed that ethanol and water extracts were the most effective^9^. The combination of betel and red betel enhanced antibacterial activity against *S. aureus*, *S. epidermidis*, and *E. coli*. According to the findings, there was no significant difference between betel and red betel against three-dimensional bacteria. Combined, they appeared antagonistic; therefore, using them separately is recommended^10^.

Antimicrobial activity and hydrothermal synergy were also liquefied by *S. platensis* and lignin, and response surface methodology (RSM) was used to optimize the performance of the parameters. Consequently, the liquefaction of *S. platensis* and lignin has an inhibitory effect, and the synergistic effect positively impacts the quality of bio-oil and higher heating value (HHV) and energy^11^.

The antibacterial effects and preservation of plant extracts and essential oils have been the subject of extensive study. Despite this, no precise research has been conducted on the topic of this study. In this study, we aimed to investigate the synergistic antibacterial effects of *Areca nut*’s fruit and *Punica granatum* L.’s peel (extracted with water, methanol, and ethanol solvents) on some of the most important bacteria that cause spoilage and poisoning in food (*E. coli*, *Salmonella*, and *S. aureus*) in response to consumer requests to improve the health of food, increase the use of natural preservatives, and extend food shelf life. This research also sought to replace natural preservatives in the food industry with conventional synthetic preservatives that cause poisoning and spoilage. In addition, using unused and inexpensive plant and natural material byproducts in producing these materials and considering the issue of sustainable development presented a challenge for this study. Therefore, introducing and selecting the most effective compound as a natural preservative for the food industry was the basis of this research.

## Materials and methods

Gentamicin antibiogram disc 10 μg, Penicillin antibiogram disc 10 μg, Mueller Hinton Agar, Mueller Hinton Broth, Salmonella Shigella (SS) Agar, Physiological serum, PBS (Phosphate buffered saline), 0.5 McFarland Turbidity Standard, methanol, ethanol was purchased from Merck (Germany). Three kettles, including ATCC 25,922 *E. coli*, ATCC 6538 *S. aureus*, and ATCC 9270 *Salmonella*, were prepared from the microbial section of Pasteur institute of Iran and transferred to the laboratory for testing.

## Collection, identification and preparation of plants

Plant materials (*Punica granatum* L. and *Areca nut*) were prepared from Hakim Razi Azar company of Iran and transferred to the pharmacognosy department of the pharmacy School of Tabriz. After identifying the species, the *Areca nut*’s fruit and *Punica granatum* L.’s peel was separated, washed, air-dried at room temperature, and ground with an electric mill. It was then strained through a 40-mesh sieve and stored in a brown glass at 4 °C.

### Extraction

The maceration method was used to extract 40 g of each plant per 1000 ml of 80% ethanol, 80% methanol, and distilled water at 25 °C for 48 h^12,13^. Extracts were filtered with Whatman filter paper (no. 4) to remove plant residues, then concentrated to one-fifth of the original volume at 45 °C using a rotary evaporator, and then dried at 50 °C in an oven. Before and after drying, the extracts’ dry weight was measured, and they were stored in sterile containers at 4 °C.

### MIC and MBC

A pure culture has a suspension of approximately 0.5. To achieve turbidity of 1 × 106, McFarland was prepared in Mueller–Hinton broth and diluted at 1:100^14^. Different dilutions were prepared in the broth medium from the sterilized extracts using a needle filter with a pore diameter of 0.22 microns, then 100 µL of different dilutions of the extract; i.e., 100 µL of bacterial suspension were poured into 96 polystyrene plates. Also, wells containing 200 μl of broth medium served as a negative control, whereas wells containing culture medium and bacteria were employed as a positive control. In addition, several wells containing 100 μl of medium and 100 μl of each dilution were considered turbidity controls. This test was repeated three times for every bacterium. The plates were then covered to prevent evaporation and incubated at 37 °C for 24 h. After 24 h, Alizairder measured the turbidity at 630 nm.

MIC was considered the extract’s lowest concentration, which reduced turbidity by 90% compared to the control group. The data’s significance level and statistical analysis (one-way analysis of variance and Tukey test) were deemed to be 0.5. The minimum bactericidal concentration of the extracts was determined by the pour plate method. To this end, samples from each well where no bacterial growth was observed were cultured on Müller Hinton agar medium and incubated at 37 °C for 24 h. The cultured plates were examined for bacterial growth. MBC was considered the lowest concentration in which no bacterial growth was observed^15,16^.

### Antibacterial interaction of combined extracts of *Punica granatum* L. and *Areca nut*

The checkerboard titration method was used to evaluate the synergistic effect of aqueous, ethanolic, and methanolic extracts of *Punica granatum* L. and *Areca nut* based on the Fractional Inhibitory Concentration (FIC). The results of this test were expressed as FIC (Eq. 1) in accordance with the MIC.

1 $${\text{FIC}} = \frac{{MIC_{in \,combination} }}{{MIC_{alone} }}$$

The Fractional Inhibitory Concentration Index (FICI) was calculated using the total FIC of every antibacterial agent. Indeed, FICI < 0.5, 0.5 < FICI < 1 and FICI > 1 indicate a synergistic effect, an additive or neutral effect, and an antagonism effect (reduction effect), respectively^17,18^.

First, two successive *Punica granatum* L. and *Areca nut* concentrations were prepared separately. In each row (from left to right), 95 μl of 100 mg/ml to 0.78 mg/ml of *Punica granatum* L. was poured. Similarly, 95 μl of from 100 to 0.78 mg/ml of *Areca nut* extract was poured from top to bottom in each column. In our final microplate, each of the concentrations of one extract was mixed with all the concentrations of the other extract.

Afterward, 10 ml of a bacterial suspension containing 107 CFU/ml was added to each well. Four wells containing 190 μl of Müller-Hinton agar medium and 10 μl of the bacterial suspension were also considered positive control.

The contents of each well were thoroughly mixed and incubated at 37 °C for 24 h. Then, 5 μl of a 5 mg/ml triphenyl tetrazolium chloride reagent was added to each microplate well and re-incubated at 37 °C for 3 h^19^.

To determine the minimum bactericidal concentration of the extracts, samples were taken from each well in which no bacterial growth was observed, cultured on Müller Hinton agar medium for 24 h at 37 °C, and then incubated at 37 °C. Finally, the plates were examined for bacterial growth. We confirm that all methods were performed in accordance with the relevant guidelines/regulations/legislation.

### Response surface methodology

The most significant objective of this research was to optimize the antibacterial synergistic effect of *Punica granatum* L. and *Areca nut* extract using the response surface method (RSM). RSM is one of the optimization techniques based on a collection of mathematical and statistical techniques. It optimizes a process associated with optimization, reduces simulation expenses, and predicts the optimization process^20^. In the current study, Design-Expert^®^ (v. 7) software based on the Box-Behnken was used to evaluate the effect of independent variables on response performance and to predict the optimal response using RSM. This method selected four independent factors to investigate the antibacterial synergistic effect of *Punica granatum* L. and *Areca nut* extract. Table 1 lists the variables and their levels.

**Table 1:** The factors and levels of the input variables.

Factors	Levels		
	− 1	0	+ 1
A: Extrtacts Conc.(mg/mL)	1	10	100
B: Bacteria	*Salmonella enterica*	*Escherichia coli*	*Staphylococcus aureus*
C: Extract	*Punica granatum* L	*Areca nut*	*Punica granatum* L. /*Areca nut*
D: Solvent	H_2_O	C_2_H_5_OH	CH_3_OH

## Results and discussion

### MIC and MBC

The MIC and MBC of *Areca nut* and *Punica granatum* L. extract against *E. coli*, *S. aureus*, and *Salmonella* are presented in Supplementary Tables 1 and 2, respectively. Consequently, it can be concluded that the *Punica granatum* L. aqueous extract had the lowest MIC against *E. coli* and that the aqueous extract of *Areca nut* exhibited the highest MIC against *Salmonella*. Also, the ethanolic extract of *Punica granatum* L. exhibited the lowest MBC against *Salmonella* (1.56 mg/ml).

### Extract FIC results

Table 2 displays the results of the FIC. It was observed that the MIC of the combined methanolic extract of *Punica granatum* L. against *S. aureus* was greater than the MIC of the single extract and was equal to 12.5 mg/ml; however, the MIC of the combined methanolic extract of *Areca nut* and aqueous extract of *Punica granatum* L. was 3.12 mg/ml. The results were identical for both the single and combined forms of *E. coli* (0.78 mg/ml) for the aqueous extract of *Punica granatum* L. According to the FIC index, the ethanolic extract of P.G.L.A.N (*Punica granatum* L. and *Areca nut* combined) exhibited a synergistic effect against *S. aureus* and *Salmonella* but an additive effect against *E. coli*. In addition, the aqueous extract of P.G.L.A.N was ineffective against *E. coli* and *S. aureus*, but it had a synergistic effect against *Salmonella*. The P.G.L.A.N methanolic extract exhibited an additive effect against *E. coli* and *Salmonella*.

**Table 2:** Results of FIC index.

Bacterium	Extracts	MIC (μg/mL)		ΣFIC		Antibacterial interaction
		Single	Combined	*Punica granatum* L./*Areca nut*	Index	
E. coli	P.G.L./A.N. (H_2_O)	0.78/6.25	0.78/0.19	1/0.03	1.03	Ineffective
	P.G.L./A.N. (CH_3_OH)	6.25/3.12	0.63/1.56	0.1/0.5	0.6	Additive effect
	P.G.L./A.N. (C_2_H_5_OH)	1.563.12	0.78/0.16	0.5/0.05	0.55	Additive effect
Salmonella	P.G.L./A.N. (H_2_O)	3.12/12.5	1.56/3.12	0.5/0.25	0.75	Additive effect
	P.G.L./A.N. (CH_3_OH)	3.12/6.25	0.39/3.12	0.125/0.5	0.625	Additive effect
	P.G.L./A.N. (C_2_H_5_OH)	1.56/3.12	0.11/0.19	0.06/0.06	0.12	Synergistic effect
S. aureus	P.G.L./A.N. (H_2_O)	3.12/6.25	3.12/3.12	1/0.5	1.5	Ineffective
	P.G.L./A.N. (CH_3_OH)	6.25/3.12	12.5/3.12	2/1	3	Ineffective
	P.G.L./A.N. (C_2_H_5_OH)	1.56 /6.25	0.06/0.25	0.04/0.04	0.08	Synergistic effect

### Response surface methodology

Following the experiment design, 29 experiments were provided using the software (Table 3).

**Table 3:** Box—Behnken design with RSM results.

Run	A:Extracts Conc. mg/mL	B: Bacteria	C:Extracts	D:Solvent	Y(Inhibitory zone diameter/ mm)
1	0	0	1	− 1	13.22
2	− 1	1	0	0	2.71
3	0	− 1	0	− 1	0.17
4	0	− 1	0	1	6.5
5	0	0	0	0	10
6	0	− 1	− 1	0	14
7	0	− 1	1	0	14.5
8	− 1	0	0	1	1.54
9	0	1	0	1	6
10	0	0	0	0	10
11	1	0	1	0	21.5
12	− 1	0	0	− 1	3.76
13	1	1	0	0	11
14	1	− 1	0	0	10
15	− 1	− 1	0	0	2.5
16	1	0	0	1	15.76
17	0	1	1	0	13.5
18	0	1	0	− 1	1.5
19	0	0	0	0	10
20	0	0	0	0	10
21	0	0	1	1	13.5
22	0	0	0	0	10
23	0	0	− 1	1	19
24	0	1	− 1	0	16.5
25	− 1	0	1	0	13
26	1	0	1	0	22.5
27	− 1	0	− 1	0	14.5
28	0	0	− 1	− 1	13.1
29	1	0	0	− 1	5.56

Equation (2) presents the final equation in terms of coding factors. In this equation, Y denotes the inhibitory zone diameter (mm). The coefficients of A, B, C, and D parameters were derived from the regression of linear effects; the coefficients of interaction parameters (AB, AC, AD, BC, BD, CD) were obtained from the regression of the interaction effects of the parameters; and the coefficients of A2, B2, C2, and D2 were inferred from the regression of power effects 2.

2 $${\text{Y }} = + 10.00 + 4.03*{\text{ A}} + 0.30*{\text{ B}} - 0.87*{\text{ C}} + 2.08*{\text{ D}} + 0.20{\mkern 1mu} *{\text{A}}*{\text{ B}} + 0.13*{\text{A}}*{\text{ C}} + 3.11*{\text{A}}*{\text{ D}} - 0.88*{\text{ B}}*{\text{C}} - 0.46*{\text{ B}}*{\text{ D}} - 1.41*{\text{C}}*{\text{ D}} - 0.12*{\text{ A}}^{{\text{2}}} - 3.30*{\text{ B}}^{{\text{2}}} + 7.94*{\text{ C}}^{{\text{2}}} - 3.21*{\text{ D}}^{{\text{2}}}$$

### Analysis of variance

Using the response surface methodology, optimal conditions and the best mathematical model were determined by analyzing the results^21^. The significance level (*P* ≤ 0.05) was determined by applying analysis of variance (ANOVA) to the coefficients. The capability of the final model was investigated using software analysis. Table 4 displays the results of the ANOVA and multiple regression coefficients for the antibacterial effect. The values of F (228.74) and *p* (< 0.001) indicate that the model is significant, and the results indicate that the model can adequately explain the percentage of antibacterial effect as a function of factors. In contrast, the value of lack of fit (LOF) = 4.28 indicates a good fit with a small error margin. The regression equation can therefore be used to explain the relationship between variables and response.

**Table 4:** Results of ANOVA.

ANOVA for response surface quadratic model						
Source	Sum of	df	Mean	F	*p* value	
	Squares		Square	Value	Prob > F	
Model	979.74	14	69.98	228.74	< 0.0001	Significant
A-Extracts conc	194.49	1	194.49	635.7	< 0.0001	
B-Bacteria	1.04	1	1.04	3.41	0.0859	
C-Extracts	8.98	1	8.98	29.35	< 0.0001	
D-Solvent	52.04	1	52.04	170.1	< 0.0001	
AB	0.16	1	0.16	0.51	0.4869	
AC	0.063	1	0.063	0.2	0.6582	
AD	38.56	1	38.56	126.05	< 0.0001	
BC	3.06	1	3.06	10.01	0.0069	
BD	0.84	1	0.84	2.74	0.1203	
CD	7.9	1	7.9	25.81	0.0002	
A^2	0.09	1	0.09	0.29	0.5957	
B^2	70.6	1	70.6	230.77	< 0.0001	
C^2	409.27	1	409.27	1337.74	< 0.0001	
D^2	66.75	1	66.75	218.18	< 0.0001	
Residual	4.28	14	0.31			
Lack of fit	4.28	10	0.43			
Pure error	0	4	0			
Cor total	984.02	28				
Std. dev	0.55	R-Squared	0.9956			
Mean	10.55	Adj R-Squared	0.9913			
C.V. %	5.25	Pred R-Squared	0.9749			
PRESS	24.67	Adeq Pprecision	55.136			

According to the *p* value among the independent variables, A, C, D, AD, BC, CD, B^2^, C^2^, and D^2^ were statistically significant, whereas B, AB, AC, and BD were not (p > 0.05). Moreover, the R^2^ value in the regression equation was 0.9956, indicating that the selection factors could account for 99.56% of the experimental results. Therefore, the antibacterial effect can be predicted using the regression model. Likewise, the output of RPred.2 and R^2^Adj. This model exhibits a high score, demonstrates the best fit between the model and the results, and accurately predicts the desired response. In conjunction with these outcomes, a relatively low CV = 5.25 for the dependent variables demonstrates the accuracy of the measurements and the dependability of the tests. Low press = 24.67 and SD = 0.55 indicate that the model is consistent with the experimental findings.

### Validation of results

**Figure 1:**
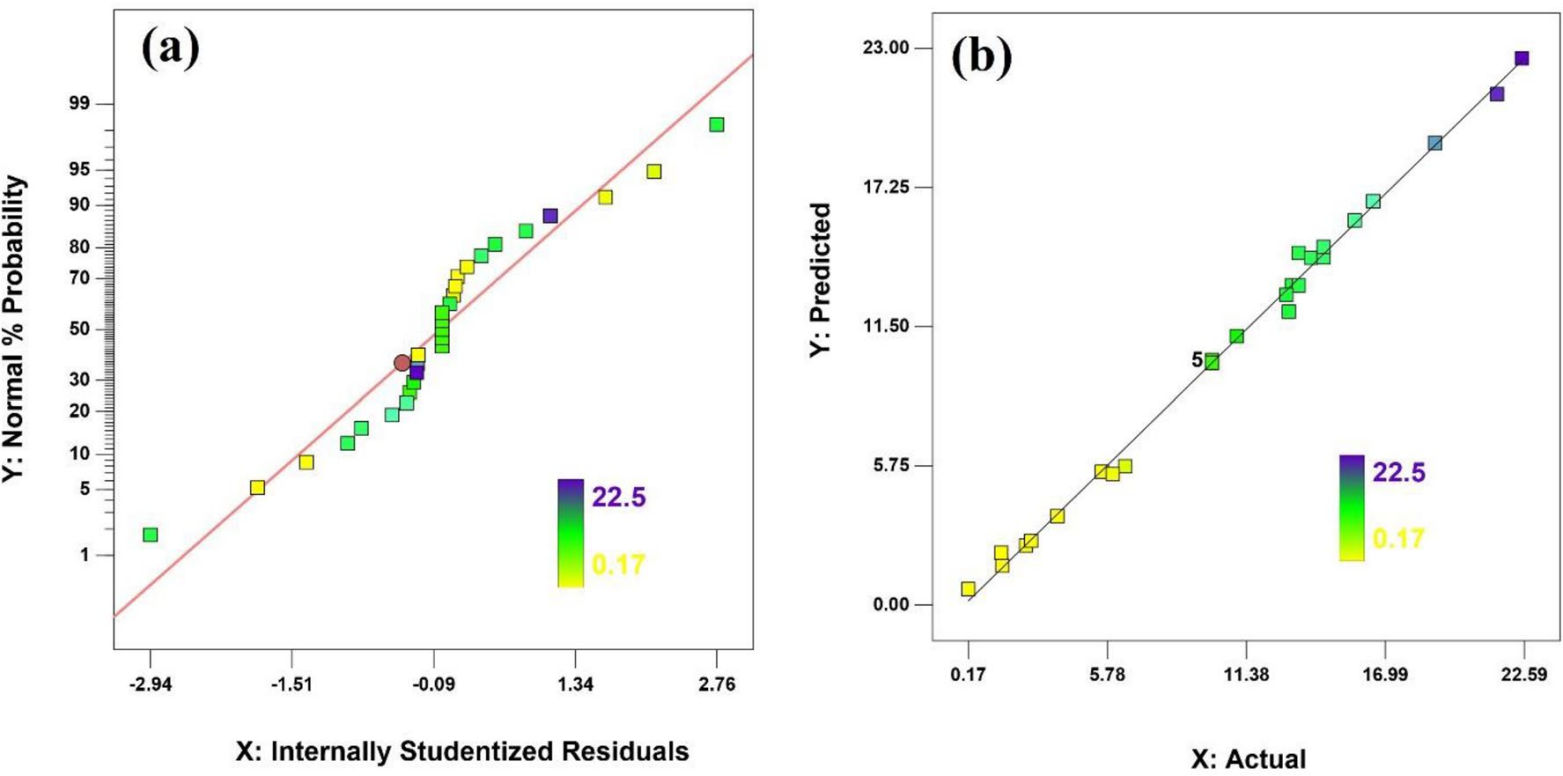
(**a**) Normal plot distributions of the residuals, (**b**) Predicted versus actual.

Figure 1 depicts normal plot distributions of the residuals and Predicted versus actual plots for inhibitory zone diameter of extracts to examine the validity of the resulting studies. The diagram of the model’s Internally Studentized Residuals is shown in Fig. 1a. In this diagram, no abnormal points or trends can be rejected based on the assumption of residual independence. Also presented in Fig. 1b is a plot of actual values versus predicted values. A normal and proper distribution of points around the straight line indicates a normal distribution of errors. Consequently, since the errors are normally distributed, the model is deemed valid, and the predicted responses correspond to the actual values^22^.

### Response of 3D plots

**Figure 2:**
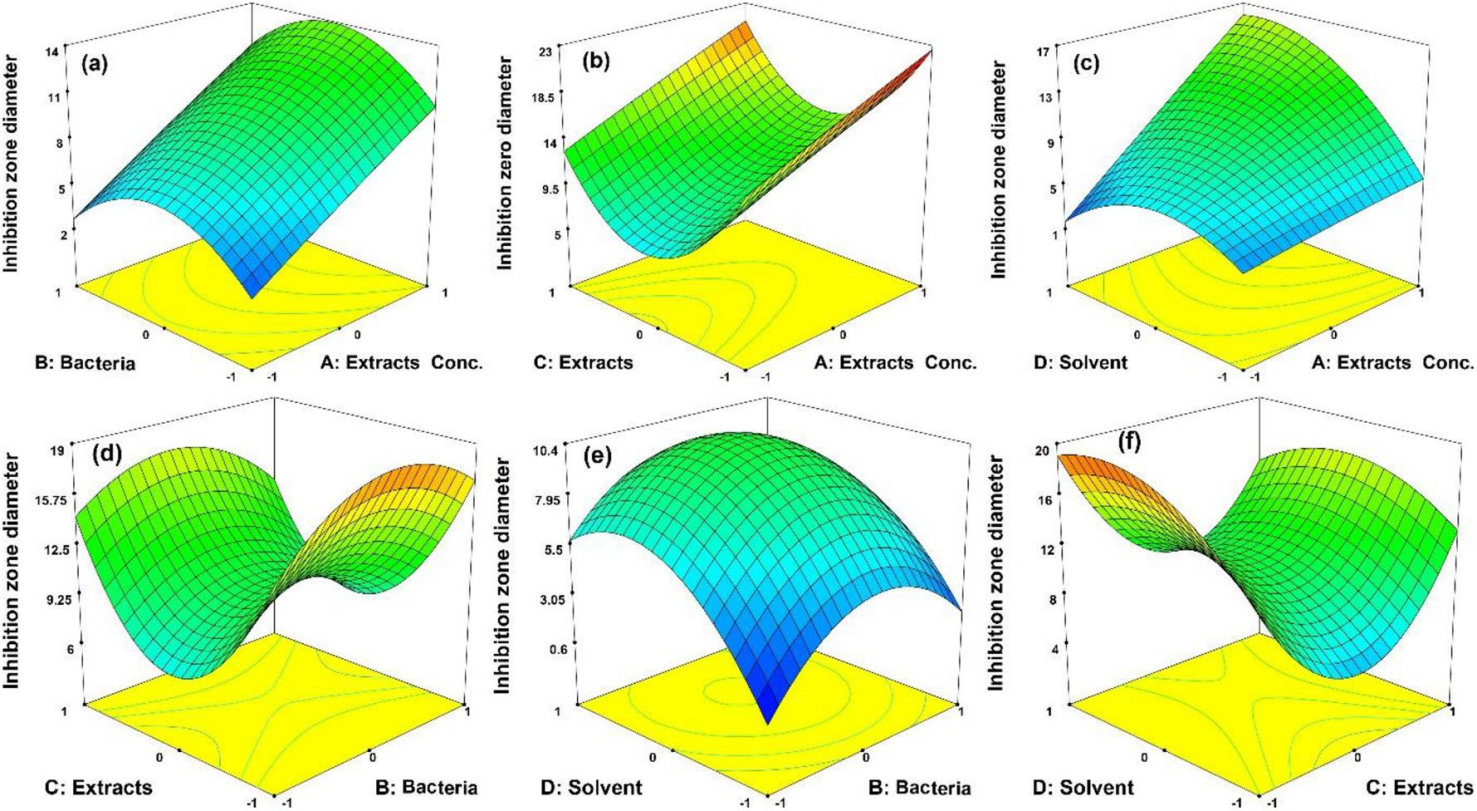
3D plots of interaction between (**a**) Bacteria and Extract concentration, (**b**) Extracts and Extract concentration, (**c**) Solvents and Extract concentration, (**d**) Extracts and Bacteria, (**e**) solvents and Bacteria, (**f**) solvents and Extracts in inhibitory zone diameter of antibacterial activity of combined *Punica granatum* L. and *Areca nut*.

The corresponding three-dimensional plots are presented in this section to study the independent parameters’ effect on the response. The interaction between extract concentration and bacteria type is depicted in Fig. 2a. As shown, 100 mg/ml of *Areca nut* ethanolic extract had the greatest inhibitory zone diameter against *E. coli*. In fact, increasing the concentration of *Areca nut* extract increases the amount of the extract’s active antibacterial substance, which inhibits the growth of bacteria. Spore production in Gram-negative bacilli and the extreme pathogenicity and antibiotic resistance of *Staphylococcus* bacteria had the greatest impact on *E. coli*^23–25^.

The interaction between extract concentration and extract type has the greatest effect on the diameter of the inhibitory zone at a concentration of 100 mg/ml in combined ethanolic extracts, as shown in Fig. 2b. The presence of an active antibacterial substance in the medium promotes and inhibits bacterial growth. Despite the presence of polar and non-polar components in the extracts, the non-polar and polar components have strengthened each other through a synergistic effect. As shown in Fig. 2c, the interaction effect of extract concentration and type of solvent reveals that 100 mg/ml of *Areca nut* ethanolic extract produces the greatest growth inhibition zone diameter effect. Due to the tendency of extracts to be non-polar and their complete solubility in ethanol, the penetration of antibacterial extract against bacteria in ethanol increases as the concentration of the extract increases. As illustrated in Fig. 2d, the interaction between bacterial and extract types significantly affected inhibitory zone diameter. Furthermore, 10 mg/ml of P.G.L.A.N extract in ethanol produces the largest diameter of the inhibition zone against *S. aureus*. Due to the polar and non-polar components of the extracts, the non-polar and polar components reinforce one another in the synergistic process. Interaction results of bacterial and solvent types (Fig. 2e) revealed 10 mg/L of *Areca nut*. Ethanolic extracts have the greatest effect on inhibition zone diameter against *E. coli*. According to Fig. 2f, .P.G.L.A.N. extracts in ethanol exhibited the greatest inhibition zone diameter in the interaction of extract type and solvent type against *E. coli* bacteria. Both *Punica granatum* L. and *Areca nut* extracts contain polar and non-polar components. In a synergistic process, a combination of extracts strengthens both the non-polar and polar components of each other. Due to the complete solubility and penetrability of the extracts, the ethanolic solvent has a greater antibacterial effect^26,27^.

### Optimization

The optimal conditions were determined to be 100 mg/ml of P.G.L.A.N ethanolic extracts against *E. coli* with a 23.48 mm inhibitory zone diameter. Moreover, the predicted diameter of the inhibitory zone under optimal conditions was approximately 23.76 mm. Consequently, the consistency between experimental and predicted results validated the model’s accuracy.

## Conclusion

This study investigated the synergistic effect of *Areca nut*, and *Punica granatum* L. extract in different concentrations of ethanol, methanol, and water against *E. coli*, *S. aureus*, and *Salmonella*. The results showed that ethanol was superior in the disc diffusion method, followed by methanol and water. Second, the extracts had the greatest effect on Gram-negative bacteria, particularly *E. coli*, based on a strong correlation between the values predicted by the RSM model and the experimental results. In addition, the results of the MIC and MBC tests support this conclusion.

## Supplementary Information

Supplementary Information.

## Data Availability

Data will be available on request to corresponding or first author.
