# Supplementary material for: Optimization of synergic antibacterial activity of Punica granatum L. and Areca nut (P.G.L.A.N) extracts through response surface methodology
Source: Sci Rep. 2023 Apr 13;13:6098. doi: 10.1038/s41598-023-32900-1 (PMC10102241; doi:10.1038/s41598-023-32900-1)
Supplement: Supplementary file 1 — Supplementary Information. [file 41598_2023_32900_MOESM1_ESM.docx]

Table 1s. Results of MIC and MIC (mg/ml) of Areca nut

| Solvents | Staphylococcus aureus | | Salmonella enterica | | Escherichia coli | |
| --- | --- | --- | --- | --- | --- | --- |
|  | MBC | MIC | MBC | MIC | MBC | MIC |
| Water | 12.5 | 6.25 | 25 | 12.5 | 12.5 | 6.25 |
| Ethanol | 6.25 | 6.25 | 12.56 | 3.12 | 12.5 | 3.12 |
| Methanol | 6.25 | 3.12 | 12.56 | 6.25 | 3.12 | 3.12 |

Table 2s.Results of MIC and MIC(mg/ml) of Punica granatum L.

| Solvents | Staphylococcus aureus | | Salmonella enterica | | Escherichia coli | |
| --- | --- | --- | --- | --- | --- | --- |
|  | MBC | MIC | MBC | MIC | MBC | MIC |
| Water | 6.25 | 3.12 | 3.12 | 3.12 | 3.12 | 0.78 |
| Ethanol | 3.12 | 1.56 | 1.56 | 1.56 | 6.25 | 1.56 |
| Methanol | 12.5 | 6.25 | 6.25 | 3.12 | 6.25 | 6.25 |
